# Understanding racial bias through electroencephalography

**DOI:** 10.1186/s40359-023-01125-2

**Published:** 2023-03-27

**Authors:** Mirella Manfredi, William E. Comfort, Lucas M. Marques, Gabriel G. Rego, Julia H. Egito, Ruth L. Romero, Paulo S. Boggio

**Affiliations:** 1 Department of Psychology, University of Zurich, Zurich, Switzerland; 2 Jacobs Center for Productive Youth Development, University of Zurich, Zurich, Switzerland; 3 Social and Cognitive Neuroscience Laboratory, Developmental Disorders Program, Centerfor Health and Biological Sciences, Mackenzie Presbyterian University, Sao Paulo, Brazil; 4 Institute of Physical Medicine and Rehabilitation, Clinical Hospital, University of Sao Paulo Medical School, São Paulo, Brazil

**Keywords:** Event-related potentials, Social neuroscience, Racial prejudice, Face recognition memory, Implicit prejudice, Empathy to pain

## Abstract

Research on racial bias in social and cognitive psychology has focused on automatic cognitive processes such as categorisation or stereotyping. Neuroimaging has revealed differences in the neural circuit when processing social information about one’s own or another’s ethnicity. This review investigates the influence of racial bias on human behaviour by reviewing studies that examined changes in neural circuitry (i.e. ERP responses) during automatic and controlled processes elicited by specific tasks. This systematic analysis of specific ERP components across different studies provides a greater understanding of how social contexts are perceived and become associated with specific stereotypes and behavioural predictions. Therefore, investigating these related cognitive and neurobiological functions can further our understanding of how racial bias affects our cognition more generally and guide more effective programs and policies aimed at its mitigation.

## Understanding racial bias through electroencephalography

The year 2020 was marked by large anti-racism demonstrations in many western countries and a broader discussion of racial bias and discrimination in the media. Although these demonstrations gained media attention due to George Floyd’s death, they are only the most recent manifestation of a continuous and enduring struggle to address social inequalities between racial groups. In addition to adopting policy measures targeting these inequalities, an essential part of this broad movement has been the role of scientific investigations to identify the cognitive aspects involved in racial bias, racial prejudice and racial stereotypes. Racial bias is defined as preconceived beliefs, attitudes, and expectations about racial group members [1]. Racial prejudice is an attitude, usually negative, directed towards another ethnic group). Finally, Racial stereotypes are a broad group generalisations that tend to disproportionately position some groups as better than others [2]. Although the terms are often used interchangeably in the scientific literature, in this review we will mainly use the term racial bias.

Research on racial bias in social and cognitive psychology has focused on automatic cognitive processes such as categorisation or stereotyping [3, 4]. More recently, neuroimaging has revealed differences in the activation of brain regions, including amygdala, anterior cingulate cortex (ACC) and dorsolateral and ventrolateral prefrontal cortex, when processing social information about one’s own or another’s ethnicity. These brain areas are also implicated in related cognitive functions such as face detection, affective evaluation and behavioural regulation [5]. The investigation of these related cognitive and neurobiological functions can further our understanding of how racial bias affects our cognition more generally and guide more effective programs and policies aimed at its mitigation, as suggested by Denson and Chang, [6]. They demonstrate that programs aimed at greater racial or ethnic diversity in education can reduce racial bias.

One of the main difficulties in this field of research has frequently been to consistently identify prejudice or bias through explicit instruments such as questionnaires or scales, for two reasons: participants may modulate their behaviour not to be seen as prejudiced given the sensitive content of the topic [7], and because of the effect of implicit prejudices and stereotypes. These phenomena might include negative attitudes an individual holds towards a social group in the absence of conscious awareness, which are resistant to detection in behavioural tasks and may negatively influence an individual’s perception and behaviour in situations of low cognitive control such as quick or automatic decisions (e.g., [7, 8]).

Given this, the adoption of techniques from the neurosciences has been of great value for identifying patterns in brain activity associated with racial bias that do not require explicit responses. Of particular relevance are those techniques that rely on brain electrophysiology recordings, such as electroencephalography (EEG) and magnetoencephalography (MEG). EEG has been consistently used to investigate temporal aspects in socially-relevant information’s perceptual and semantic processing. Because accurate temporal resolution is crucial for pinpointing and analysing the different processes involved in racial bias, event-related potentials (ERPs) measured via electroencephalography (EEG) are particularly well-suited to this aim. ERPs reflect the postsynaptic activity of an extensive set of neurons activated in close temporal proximity and can be recorded by placing several electrodes on the surface of the participant’s head [9]. The electric wave detected in the ERP is composed by the summing of subjacent components, where each component represent a brain activity associated with a specific cognitive process and which is characterised by: (a) *latency*, at what moment in time they occur after the onset of a specific stimulus; (b) *direction*, either positive- or negative-going; (c) *amplitude* of the wave, which represents the demand of brain activity and indirectly the level of cognitive engagement of a specific process; (d) *localisation* over the scalp, indicating in what electrode region (from an international measurement system) the component is usually detected; and (e) *source*, i.e., the probable cortical brain region (or regions) originating the signal [10]. Differences in ERP component amplitude usually indicate distinct cognitive demand levels of cognitive processes (e.g., attention, memory, motivation, etc.) or distinct cognitive mechanisms involved in information processing. ERP latency indicates the specific moment where this process occurs after stimulus appearance.

### ERP components involved in racial bias

A significant body of literature has employed an ERP design to investigate electrophysiological responses associated with the racial bias process phases. These studies identified the following ERP components: N1/N170, P2, N2/N250, P300, ERN, Pe, N400 and LPP.

The N1 is the first negative deflection on posterior scalp regions, which can be observed in response to any visual information. The N1 has a peak latency of 130–200 ms. The N170 is an early component that has been associated with the perceptual processing of faces and peaks on average at about 160–170 ms for these stimuli. Various kinds of information can also be extracted quickly and efficiently from the face to categorise the person’s gender, facial expression, ethnic origin, and gaze direction [11]. The anterior N2/N250 peaks approximately 250 ms after stimulus presentation and appears to index selective attention to specific stimulus features (e.g., colour, shape, form; [12]. An N2 effect has been observed for stimuli containing conflicting features, as in the flanker and Stroop paradigms.

Similarly to N2, the MFN is sensitive to conflict, but it appears to be modulated by expectations in context: when most trials within a block are congruent, the MFN is larger for incongruent trials [13].

The P300 usually peaks between 300 and 500 ms after stimulus onset and is classically associated with task relevance. The P300 is larger for infrequent target stimuli and is strongly modulated by attention [14]. The P300 is also sensitive to emotional information (both pleasant and unpleasant stimuli), suggesting the P300 may reflect a general motivation [15, 16].

The error-related negativity (ERN) reflects error detection in many tasks. It is sensitive to different types of sensory information, such as auditory, visual, and somatosensory. This component typically occurs at the moment of the erroneous button press and peaks around 100 ms later. This component also represents a self-monitoring mechanism that allows volunteers to improve their cognitive control over prepotent responses continually.

The error positivity (Pe) component usually follows the ERN. It is usually larger in response to errors that the participant reports compared with those that are not reported. This component has been associated with an affective response to the error, awareness of the error, or adapting response strategies after error detection.

A significant body of literature has investigated semantic processing by analysing the N400, a negative event-related brain potential that peaks roughly 400 ms after the onset of a stimulus [17, 18]. The N400 peaks roughly 400 ms after the onset of a stimulus and reflects the spreading activation in the access of semantic information by a stimulus in relation to its preceding context [18]. This component is sensitive to semantic violations related to a large array of meaningful information. The late positivity (LP/P600) is a slow late positive shift that typically onsets around 500 ms after the onset of a stimulus. It has been related to a process of reanalysis of the incongruent situation produced by inconsistent meaning [19–21], particularly when the incoming information disconfirms predictions created by a preceding context [22].

### Aim of the present review

When studying racial bias, the ERP technique allows us to understand further the role of subjacent cognitive processes (e.g., attention, memory) involved in the detection, categorisation, and reaction to stimuli involving same- or other-race. It also enlightens us about the correlation of the ERP components to other psychological aspects such as personality traits or explicit prejudice/stereotyping. Besides, understanding when and how social information is processed using ERP could help us make inferences about brain processes dynamics involved in racial bias and social cognition overall [5].

This review investigates how racial bias, implicit or explicit, influences human behaviour. To this aim, we identify the social and cognitive neuroscience topics that adopted ERP and the characteristics of the detected ERP components. The investigation of specific ERP components allows us to analyse changes in neural circuitry during automatic and controlled processes elicited by specific tasks. Therefore, a systematic analysis of specific ERP components across different studies provides a greater understanding of how social contexts are perceived and become associated with specific stereotypes and behavioral predictions.

Based on the available studies on this topic, we will break the discussion down into the following main sections: (i) *Face recognition*; (ii) *Implicit bias*, including studies addressing implicit prejudice and stereotyping; and (iii) *Pain empathy*.

### Face recognition

A widely studied topic in neuroscience of racial bias is detecting and recognising same- or other-race faces. Two phenomena have been observed: the “Other-Race Effect” (ORE), where people identify faces of individuals from own-race easier than other-race faces (e.g., [23–27], and the “Other-Race Advantage” (ORA), where people are faster to categorise other- compared to same-race faces [28, 29].

In the literature, several explanations have been proposed regarding the mechanisms that differentiate the ORA from the ORE. According to the model of Valentine and colleagues [30], faces are encoded as nodes in an *n*-dimensional space in which the distance between two nodes is inversely related to their subjective similarity. The classification of a face results from the sum of the total activation of all neighbouring nodes, and increasing the total activation of nodes in a given group leads to faster classification. Thus, the higher homogeneity of other-race faces compared to own-race faces in representational space could lead to an easier spread of activation among other-race faces. This phenomenon could explain the faster and more accurate classification of other-race faces than own-race faces according to race (ORA) and the slower and less accurate identification of individual other-race faces than own-race faces (ORE).

In addition, the literature has shown constraining results in neurocognitive responses to these phenomena, which will be discussed in the following sections.

#### Early perceptual processing

Face detection and recognition is a fast and automatic feature in humans. Thus several early latency ERP components (i.e., occurring before 200 ms) are implied in this process, such as P1, N170, P2 and N2 [28, 31, 32], where some studies also report findings with middle or late components such as N250 or P3 (e.g., [33–35]).

With regards to racial features detection, there are contradictory findings. Some studies did not find a modulation of face-specific N170 response to same- or other-race faces (e.g., [28, 31, 32]), whereas other studies found differences but in both directions (i.e., larger for other-race, or larger for own-race, [36, 37].

The conflicting N170 responses across studies suggest that this ERP response is sensitive to the context, material and demands of the experiment task. Further investigation would be useful to clarify the interaction between these elements and the effects on the N170 response.

Studies conducted on the ORE frequently manipulate facial features such as shape and colour to explore how processing of SR and OR faces occurs. Brebner et al. [24] observed an increase in N170 amplitude for OR faces linked to changes along a gradient of skin colour (from light-skinned to dark-skinned), with a significantly greater N170 amplitude in response to darker-skinned OR faces than lighter-skinned faces. Similar increases in amplitude have been observed for both the N170 [27, 33, 38–41], and other early components related to attentional modulation (i.e., N250, N200) and memory encoding (i.e., LPP) in response to the perception of OR compared to SR faces [26, 33–35, 42–45]. Furthermore, these ERP responses (e.g. N200) were modulated by colour and facial structure and correlated with subsequent memory performance. In two studies [40, 41], the repetition suppression paradigm was used to directly test the effect of the other race. Using the EEG, they found stronger individuation processing for SR and stronger categorization processing for OR, indexed by the N170 and P2 components. Given this evidence, these results suggest that racial differences in skin colour and facial structure are detected during the encoding of unfamiliar faces and that the detection of these features may be a critical factor in the ability to remember them later [42].

#### Face recognition memory

Several studies have found better memory performance for same-race (SR) compared other-race faces [46–50]. In 2011 Lucas et al. investigated neural mechanisms of this memory bias by recording event-related potentials during a same-race (SR), and other-race (OR) faces memory task. They found late positive responses (LP) to memory recall for SR faces and, to a lesser extent, for OR faces. However, they also found greater N200 and P2 responses to OR faces with race-atypical features than faces that were race-stereotypical. According to the authors, these earlier potentials index the processing of unique facial information, crucial for remembering a face. This phenomenon, defined as Individuation, is usually greater for SR faces but lower and less reliable for OR faces. However, this recent evidence suggests that it is more frequent for OR faces that appear less stereotypical. In Herzmann et al. [51] analysed the neural responses during an associative-memory task, in which Caucasian and East Asian participants learned and recognised own-race and other-race faces. Participants showed a greater positivity to own-race faces, indicating more detailed retrieval of own-race faces and suggesting a better memory performance in recognising own-race faces than other race faces.

As previous studies suggest specific deficits in perception and subsequent memory of faces in older adults, some recent studies explored the impact of ageing on the processing and memorisation of young/old and White/Asian faces. Considering ORE, Komes et al. [25] tested older adults grouped according to their level of performance on tests of cognitive ability (high- and low-performing). Both groups showed a clear ORE for young faces from their racial ingroup (White), with significantly larger N170 amplitudes in response to memorising young Asian faces but not old Asian faces. However, group differences emerged independently of facial ethnicity: while low-performing subjects showed a lateralised N170 to the right, high-performing subjects showed a more bilateral response. The authors argue that this surprising result may be due to a compensatory mechanism counteracting age-related decline in face perception enabling more efficient encoding into memory in high performers. This result is consistent with two previous studies [44, 52] and reveals the interaction between two perceptual outgroups (specifically Young and Asian) leading to a minimisation of the salience of ethnic facial cues with age and a greater perceptual marker for young (outgroup) faces.

More recently, Proverbio and De Gabriele, [53] conducted a similar study in which participants categorised three images as straight or inverted following the presentation of Caucasian and African faces of infants and adults. They observed larger N2 amplitudes originating in frontal structures in response to infants’ faces compared to adults, with no effects attributable to race. However, an increase in P3 amplitude was observed in response to Caucasian faces compared to African faces, for adult faces only. These results confirm the visual primacy of age-related cues of facial perception over cues related to ethnicity or race.

Taken together, these results indicate that the ORE may be a generalised phenomenon which is applicable to other social outgroups beyond racial ethnicity, and that repeated exposure to a specific outgroup may lead to greater expertise in face recognition for individuals from that outgroup.

Facial cues for gender categorisation also appear to modulate the effect of the ORE by minimising the salience of face ethnicity [52], highlighting the task-specific nature of face recognition [54] and the secondary priority of facial cues for ethnicity compared to cues of gender, age and emotional expression. Wolff et al. [55] observed an own-gender bias for both male and female subjects, with increased performance in recognising familiar faces from one’s gender compared to the opposite gender. This own-gender bias was also reflected in the electrophysiological markers associated with initial face processing, with significantly smaller P2 and N2 amplitudes in response to processing the subject’s own face gender compared to faces of the opposite gender. In Wiese and Schweinberger study [56], male and female participants showed own-race biases, reflected in a larger N170 in memory performance. In fact, previous evidence show a correlation between between the magnitude of the N170 ethnicity effect during the learning phases and the magnitude of one's race bias at the time of the test (see [27]. In addition, only in female participants, better memory for own-gender faces as reflected in the N250 response. The authors concluded that since different face memory biases occur at temporally distinct stages of face processing, they would be based on different neurocognitive mechanisms.

In conclusion, the results of these studies suggest no differences in perceptual competence, but instead, the differences in ERPs reflect neural correlates of the effect of perceived social category membership on facial recognition memory. These findings align with socio-cognitive theories that highlight the significance of categorising a face as being either a member of a social ‘ingroup’ or an ‘outgroup’ [57, 58]. Consequently, bias in face memory could be caused by the fact that faces belonging to an “outgroup” are processed at a categorical level, whereas “ingroup” faces are individualised. Such individualisation would involve deeper processing and thus more accurate recognition memory.

It is interesting to note that context and changes in task demands can modulate the electrophysiological correlates of the ORE. Minor modulations by visual expertise have been observed in Asian subjects following previous experience with the majority ethnicity (White in the US), leading to smaller increases in N170 and N250 amplitudes when categorising outgroup faces which form part of the majority ethnicity [26, 42]. A more direct investigation of the effect of face expertise on N170 amplitude was conducted by Balas and Saville [59]. Essentially, they found that individuals recruited from small towns (500–1000 inhabitants) showed similar N170 amplitudes for both faces and objects compared to a more face-selective N170 amplitude for participants recruited from big cities (30,000 to 100,000 inhabitants). In addition, small-town participants were significantly less accurate at memorising previously-presented faces and discriminating between a novel and unfamiliar faces. Since ORE can be influenced by visual competence [60], the individual level of visual competence seems to be the main determinant of inefficient coding of facial stimuli in a racial outgroup.

The great variety and number of faces one may interact with during education influences the neurocognitive mechanisms underlying face processing. Poor exposure to faces belonging to other categories may decrease memory and a reduced ability to discriminate between members of these categories compared to faces belonging to one’s own categories.

In conclusion, these studies on the detection and recognition of same- or other-race faces suggest that the ORE is due to differences in the processing of other-race and same-race faces at an early stage of encoding associated with the visual familiarity of the face as defined by recognition memory paradigms. The inefficient encoding of other-race faces into long-term memory is primarily due to a lack of visual expertise and/or previous experience with faces from a different ethnicity to one’s own. While an electrophysiological component at approximately 250 ms is associated with race perception and may act as a perceptually-salient “tag” for further visual processing [27], facial cues related to ethnicity (eg. skin color, eye width) can be selectively modulated by facial cues corresponding to gender and age. This evidence suggests that facial cues related to ethnicity might be secondary to more socially-relevant facial information.

### Implicit bias

Implicit bias is the unconscious attribution of particular qualities to a member from a particular social group [61]. It is based on internal schemas originating from associative learning, where social categories are associated with affects or beliefs, primarily influenced by cultural and social context and that can modify an individual’s subjective evaluation, attitude and reaction toward social objects [62–64].

There are several experimental paradigms to investigate implicit bias, such as the Weapon Identification Task (WIT; [8]), the First-Person Shooter task (FPS; [65]), the Implicit Association Test (IAT; [66]), or the Affect Misattribution procedure [67]. These paradigms investigate implicit attitudes or stereotypes through tests that require quick responses in associating or categorising a series of stimuli. Rapid responses tend to hinder the use of cognitive control, and variations in response time and accuracy may indicate the existence of biases in perception and reaction to other-race stimuli compared to ingroup stimuli. Among those tasks, IAT, WIT, and FPS were adopted to study racial bias through ERP analysis. Thus, we will briefly describe those tasks and the main findings concerning the detected electrophysiological components.

#### Weapon identification task

The Weapons Identification task (WIT) is a computer-designed paradigm where the participant must categorise an object as a weapon or a tool as quickly as possible, after seeing a face from an unknown person from the same- or other-race (a priming stimulus). In two consecutive studies, Amodio et al. [3, 68] demonstrated that enhanced control, defined as increased response time and greater accuracy, to race-biased responses were positively correlated with the Error-Related Negativity potential (ERN) amplitude. This component is usually detected after unexpected stimuli, such as errors or false alarm, and might also represent a self-monitoring mechanism. More recently, Fleming et al. [69] observed that North-American military cadets fire more quickly and accurately at targets holding a gun when paired with a Middle-Eastern male body in traditional Arabic clothing. However, this increase in firing rate was accompanied by a correspondingly greater bias toward classifying objects in the hands of these targets as a gun compared to targets from other ethnic groups. Consistent with its role in conflict monitoring, greater ERN amplitudes originating in the medial-frontal cortex were observed in the misclassification of tools associated with these targets. In conclusion, these studies showed that larger ERNs to racial responses correspond to higher levels of control during the task suggesting that race-based responses can be made despite activation of neural systems designed to detect bias and recruit controlled processing.

#### First-person shooter task

Other classical experiment is the First-Person Shooter task (FPST; [65] which is composed of a series of trials viewing different background images in which Black or White males may appear, holding a gun or a neutral object such as a wallet or cell phone. During the FPST, participants pretend to be in the police office dilemma where they must press a specific bottom to shoot armed targets, which indicate social threat, and press a different bottom not to shoot unarmed targets. Correll, Urland and Ito [70] found significantly higher P2 amplitudes in Caucasian participants in response to Black armed targets than White armed or unarmed targets. In contrast, they found larger N2 amplitudes for unarmed targets compared to armed targets and White targets compared to Black targets. These findings demonstrate that these components show a high degree of specificity: a greater P2 amplitudes seem to be associated with alertness while a higher N2 amplitude is associated with a greater inhibition. The data suggest that threat perception and conflict detection play a crucial role. According to the authors, this finding would suggest that factors that modulate the relationship between threat and race in the real world could impact racial bias.

Additionally, Xu and Inzlicht, [71] investigated the association between neural (ERN and Pe) and behavioural responses to errors in a shooting game. Participants completed a Shooter go/no-go task, which required shooting armed targets using a game gun and avoiding shooting innocent non-targets. The results showed that shooting errors elicited greater ERN and Pe amplitudes than correct shooting and that greater amplitudes of these neural responses were correlated with more accurate behavioural performance.

Overall, these studies suggest that it is possible to obtain online measures of brain response to shooting responses and that neural responses to shooting are predictive of response bias.

#### Implicit association test

Finally, the Implicit Association Test (IAT [66]); a reaction time-based measure of implicit social attitudes [72], has been one of the benchmarks for research into racial bias. Research participants may not always want to or be capable of reporting their true attitudes about socially sensitive issues. For these reasons, implicit measures that could reveal the types and strengths of evaluative associations without reliance on self-report promise to significantly advance the scientific study of attitudes.

A primary concern with most measures based on self-report, and even measures based on observable behaviour, is that participants can control their responses to obscure underlying evaluations. Thus, the ideal for an implicit measure is that it be structured to avoid the influence of control-related processes, whereby responses are based purely on automatically activated evaluations and associations.

In behavioural studies, the IAT has been employed to compare the reaction times in response to associations between stimuli and attributes, such as Black or White faces and positive- or negative-valence words. Studies employing an IAT-EEG design have explored how automatic racial bias can modulate the direction of attention, cognitive control and decision-making over time. Therefore, this instrument has been of great importance and utility in understanding racial bias processing.

The most common ERPs studied during implicit association processing are N170, N200, P300 and MFN. Ofan, Rubin and Amodio [73] explored anxiety effects on the neural processing of faces versus non-faces during a priming task, examining modulations on N170. They found that participants with higher social anxiety exhibited greater processing, reflected in a larger N170, of Black faces compared to White faces just in public conditions, such as monitoring by the experimenter. Additionally, higher N200, which often reflects monitoring of response conflict [74, 75], was observed after presenting uncommon associations stimuli, such as Black faces with Positive attributes for White participants. This higher amplitude reflects a cognitive demand to inhibit automatic responses, as the association between outgroup faces with Negative attributes [72].

In another study, Coates and Campbell [72] found that common associations on IAT resulted in a single P300 peak, while uncommon associations resulted in a sequence of two peaks. Thus, the authors argue that the reduced P300 amplitudes in response to uncommon associations may be related to a lack of certainty with respect to the decision.

In 2015, Hilgard and colleagues conducted a study investigating congruency and task switching effects in the IAT. During the IAT, the participants must switch between semantically categorising attitude objects on some trials (e.g. classifying names as Black or White) and evaluating words (as good or bad) on other trials. The authors found higher MFN amplitude on IAT blocks in response to uncommon associations (e.g. Black/Positive and White/Negative) in this study. This response was more negative for incongruent than congruent trials but more positive for switch than for no-switch trials, suggesting separable control processes are engaged by these two factors.

Overall, these studies showed that it is easier to categorise familiar stimuli such as faces from our own social or ethnic ingroup, and we may hold an implicit bias to associate positive attributes with our ingroup and negative attributes with members of other outgroups. These give rise to the significant effects commonly observed in the IAT. Based on data collected with the IAT, implicit preferences for a specific ethnic group seem to form part of human social cognition and are relatively stable over time and shared among members of that ingroup [76]. In sum, since testing implicit bias is associated with a large degree of individual variability and different levels of self-control, the methodological standardisation of investigation in this area is necessary to understand small levels of inter-subject variability in IAT-ERP studies accessing different ethnic samples. Unbiased participants may not show any differences in cognitive effort regarding their electrophysiological markers during the classification of common and uncommon associations, but we have no clear data of how unbiased subjects process stimulus associations differently. For example, it is unclear whether they have an automatic association specific to a certain ethnic outgroup or have a greater level of cognitive control. Considering all of these factors, it would be enlightening for IAT to be applied in samples of, for example, children from interracial couples or from clinical populations that are extremely sociable and have impairments in cognitive control, such as Williams Syndrome.

The present section highlighted the importance of studying the temporal organisation of the mental processes involved in implicit prejudice. Furthermore, the findings mentioned above demonstrate that electrophysiological components constitute an effective means of objectively assessing automatic preferences and personal prejudices.

The study of implicit prejudice as mapped by ERPs may help us understand conflict-monitoring processes, which form an essential function for regulating much of our everyday behaviour.

### Empathy to pain

Our understanding of the neural mechanisms involved in processing aversive stimuli such as pain has been long-established via physiological investigations [77–79]. More recently, experiments in cognitive psychology have shown that individuals report a heightened level of discomfort, either when directly stimulated with an aversive or painful stimulus or when observing the application of an aversive stimulus to others [80–84]. The perception of other individuals in painful or aversive conditions typically results in high levels of aversive self-report [81]. More recently, advances in social neuroscience have demonstrated that under both conditions of self-exposure to painful stimuli and the perception of pain stimulation in others, a common network of cortical and subcortical neural structures are recruited in the processing of aversive stimuli (see [83, 84]). Within this network, the principal cortical structures involved in aversive stimuli perception includes the Prefrontal Cortex (PFC) and the Anterior Cingulate Cortex (ACC), and the main subcortical structures include the anterior Insula (aI) and amygdala [84], also observed for different kinds of perceived pain [85, 86]. Furthermore, some studies have shown that features such as social context [80] and cultural influence [82] can modulate an individual’s empathic responses to the pain of others. So arises the question: are empathic response levels related to another individual’s pain perception or determined by ethnic differences? In the context of the current review: how can the ethnic differences observed in individuals influence the electrophysiological processing of a pain-related situation?

It has been observed that empathic neural responses to perceived pain are stronger for ingroup members than for outgroup members. In other words, social relationships influence empathy in people [87]. This phenomenon is known as racial bias in empathy (RBE).

A recent meta-analysis [88] identifies several ERP components that would be directly associated with the observation of pain in others. These components are the centre-parietal P3 component and the late positive potential. These components have been observed in studies investigating the interaction between empathy and racial bias. However, in the studies reviewed here, the early N1 and N2 components not always associated with the observation of vicarious pain, were also observed. As the authors of previous studies also note, these early components could be associated with the first ethnic group identification that precedes the empathic response.

#### Racial bias in empathy in static tasks

In 2012, Sheng & Han conducted a study to test whether perceiving a person of another race as a symbol of a racial group decreases empathy for that person. Initially, it has been observed a higher amplitude of P2 in response to Asian faces (ingroup) expressing pain than White (outgroup) painful faces. However, subsequent experiments showed that paying attention to the observed individual’s feelings of pain and including individuals of other races in one’s team increased neural responses to expressions of pain in the faces of other races, thus suppressing RBE. This finding suggests that manipulations of intergroup relations may decrease RBE-related brain activity.

More recently, Sheng et al. [89] found similar effects: higher P2 amplitudes in Chinese subjects in response to Chinese faces than White faces, even while recording during a perceptual and not a race- or empathy-based task. Participants were instructed to classify each face for their orientation and not their ethnicity (Chinese or White) or expression (painful or neutral). This study confirms Sheng and Han’s [87] finding that there is a greater response to pain to ingroup faces than outgroup faces.

In 2019, Li and Han tested the pain sensitivity of own-race and other-race faces in images with different pixel-scale definitions. It was observed that fewer painful pixels were necessary to perceive the pain expressions of same-race faces compared to faces of other races and that P2 amplitude increased faster in response to increasing the number of pain pixels of same-race faces compared to faces of other races. These results support the evidence of increased sensitivity of neural responses associated with subtle variations in pain expressions of same-race faces compared to other-race faces.

In contrast to the paradigm used by Sheng and Han [87], in the study run by Contreras-Huerta et al. [90], no physical clues were associated with group members, such that the group and outgroup members could not be distinguished solely by low-level visual features. The results showed no specific electrophysiological modulation due to ingroup manipulation for the N2 or P3 components. However, they found an increase in N1 amplitude in response to the observation of ingroup (White) individuals receiving painful stimulation compared to non-painful stimulation. Higher P3 amplitudes were elicited in response to the perception of individuals receiving painful stimulation, regardless of the ethnic group (White or Asian) at a later stage of processing. These results support a model of empathy for pain that consists of early, automatic empathic responses to people from their own race, as indexed by N1, and a subsequent general salience response to painful stimuli that does not differentiate between races associated with the P3 response.

Despite differences in early empathic responses in the two groups due to methodological differences [90], taken together, these studies demonstrate the influence of the perception of individuals in the group, leading to an increase in the magnitude of early ERP components in empathic responses to pain.

Sheng et al. [91] investigated whether the subsequent presentation of faces of different ethnic groups expressing pain can modulate electrophysiological response. Accordingly, Chinese participants were presented with adapter faces (with pain or neutral expressions) and target faces (with only pain expression) in rapid succession. The face pair could be either congruent (both faces had the same ethnicity) or incongruent (ex. White and Asian). Corroborating the previously cited studies, the authors found increased P2 amplitude relative to ingroup faces compared to outgroup faces. However, this effect was only observed when the face sequences were congruent with face ethnicity. The increase in P2/N2 occurred only when an adaptor and a target were of the same race, and these effects were observed in both Chinese and Caucasian participants. This finding suggests that perceiving pain expressions from different races may engage distinct neurocognitive processing.

#### Racial bias in empathy in dynamic tasks

Paradigms involving static tasks, such as observing images of faces expressing pain, provide important evidence and scientific insights into the influence of ethnic differences on the processing of a pain-related situation. However, everyday social interactions occur dynamically, leaving the doubt: would the same electrophysiological patterns found with the presentation of images be found with videos presenting faces of pain?

Sessa et al. [92] tested white participants performing a pain assessment task based on the videos of the faces of Whites and African individuals being touched by a needle or cotton swab. They observed an increase in the amplitude of the ERP components N2 and N3 in response to the processing of White faces in painful situations compared to situations without pain. This effect was not observed for African faces. In addition, no differences between ethnic groups were observed at later stages of processing (P3 response). Furthermore, they observed a pole of activation over the left medial frontal gyrus (MFG) for the P3 component in response to the observation of ingroup pain, while the source of P3 activation in response to the observation of outgroup stimuli was located in the TPJ. Thus, in line with the studies mentioned above, the authors concluded that earlier processing stages are more susceptible to modulation by ethnic group, as observed by the modulation of N2/N3. Later stages of processing, while still sensitive to race-related modulation, represent a greater degree of control over higher cognitive functions such as mentalising and cognitive reprocessing, as observed at P3. It is important to note that, despite using different techniques and tasks, Sheng et al. [93] findings showing TPJ recruitment during the tasks of pain discrimination are consistent with the results from Sessa et al. [92]. Together, they highlight the importance of a region in the posterior temporal cortex, including the TPJ, in the cognitive processing or inference about the mental state of others, also known as Theory of Mind (TOM, [94]).

Another interesting issue involving these studies is the relationship between the electrophysiological response of pain empathy processing and levels of empathy. These levels are typically measured through behavioural scales, such as the Interpersonal Reactivity Scale (IRI) initially designed by Davis [95]. Sessa et al. [92] found a positive correlation between white individuals’ performance on the IRI subtest “Empathic Concern” and N2/N3 amplitude when observing ingroup faces in pain situations. However, no significant correlation was observed for outgroup faces in pain situations. This result suggests that empathy levels can be predicted from the electrophysiological responses to other individuals in painful situations, but only at the early stages of processing and only for individuals from the same ethnic group.

Similarly, a study by Han et al. [96] sought to test the influence of different oral mimicry (contracted and relaxed oral musculature) on neurocognitive processing during the observation of facial imagery expressing pain in an ingroup (Asian) and outgroup (White) individuals. This design builds on classic studies testing the influence of mimicry and emotional embodiment on subsequent cognitive and emotional processing (see [97] for a review). Consistent with the literature [87, 90, 91, 93], greater amplitudes of N1, N2 and P3 were observed for faces expressing pain compared to emotionally neutral faces, with the greatest amplitudes observed in response to faces belonging to the same ethnic group.

Finally, several behavioural studies [98–100] have shown that individuals typically tend to conform to the opinion of other ingroup members in response to anxiety generated by priming to death or threat stimuli (an effect known as mortality salience or terror management). In support of this hypothesis, a Chinese study used a mortality salience induction method to investigate the effects of pain empathy in individuals from different ethnic groups [99]. In the early stages of processing, they found higher P2 amplitudes only when observing Asian individuals (ingroup) in painful situations regardless of the experimental condition (induction to mortality salience compared to the control condition). However, in subsequent processing steps, the induction of mortality salience led to an increase in the amplitude of the P3 component in response to the observation of ingroup individuals in painful situations. These results demonstrate that induction of mortality salience leads to increased empathy responses towards ingroup individuals and that this type of induction could also be involved in the electrophysiological modulation of final phase processing related to pain empathy.

Overall, these results indicate that the perception of pain expressions of different races may elicit a greater empathic response to ingroup faces than to outgroup faces. This response was observed in both early (N2, P2) and later processing stages (P3) of pain information. Furthermore, the P3 potential was more influenced by cognitive processes, such as the theory of mind, related to the activity of regions such as the TPJ and subjective measures such as the Interpersonal Reactivity Scale.

Visually witnessing ethnic individuals from the group in pain-related situations leads to more rapid recruitment of brain areas related to empathy and TOM than observing individuals from the outgroup, which may facilitate a greater degree of empathic response (Table 1).

**Table 1:** Summary of studies included in the systematic review

Topic	Subtopic	ERP	Function	Studies
Face recognition	Early perceptual processing	N170	Increase in N170 amplitudes for OR	Brebner et al. [24]
		N200	Higher N200 amplitudes to the perception/categorisation of OR compared to SR faces	Ito and Urland [43], Herrmann et al. [35], Stahl et al. [26], Herzmann et al. [42], Wiese [44]
		N250	Higher N250 ampltudes response to the perception/categorisation of OR compared to SR faces	Ito and Urland [43], Herrmann et al. [35], Herzmann et al. [42], Wiese [44]
		LPP	Higher LPP amplitudes to the perception/categorisation of OR compared to SR faces	Ito and Urland [43], Wiese [44]
	Face recognition memory	N200	Higher N200 to OR faces with race-atypical features than faces that were race-stereotypical	Lucas et al. [101]
		P2	Higher P2 to OR faces with race-atypical features than faces that were race-stereotypical	Lucas et al. [101]
Implicit bias	Weapon identification task	ERN	Higher ERN amplitude is positively related to enhanced control during WIT	[3, 68]
			Higher ERN amplitudes observed for misclassification of tools associated with these targets	Fleming et al. [69]
	First-person shooter task	P2	Higher P2 amplitudes in Caucasian in response to Black armed targets than White armed or unarmed targets	Correll, Urland and Ito [70]
		N2	Higher N2 amplitudes for unarmed targets compared to armed targets and White targets compared to Black targets	Correll, Urland and Ito [70]
		ERN	Higher ERN amplitudes related to shooting errors compared to correct shooting	Xu and Inzlicht, [71]
	Implicit association test (IAT)	N170	Higher N170 related to higher social anxiety faced with Black faces compared to White faces	Ofan, Rubin and Amodio [73]
		N200	Higher N200 when presenting uncommon associations stimuli	Ofan, Rubin and Amodio [73]
		P300	Single P300 peak observed for common associations, while two peaks observed for uncommon associations	Coates and Campbell [72]
		MFN	Higher MFN amplitude in response to uncommon associations	Hilgard et al. [13]
Empathy to Pain	Racial bias in empathy in static tasks	N1	Higher N1 amplitudes when facing ingroup pain faces, compared to outgroup faces	Contreras-Huerta et al. [90]
		P2	Higher P2 amplitudes when facing ingroup pain faces, compared to outgroup faces	Li and Han [102], Sheng and Han [87], Sheng et al. [91], Sheng et al. [89]
		P3	Higher P3 amplitudes when facing ingroup pain faces, compared to outgroup faces	Contreras-Huerta et al. [90]
	Racial bias in empathy in dynamic tasks	N2	Higher N2 amplitudes when facing ingroup pain faces, compared to outgroup faces	Sessa et al. [92]
		N3	Higher N3 amplitudes when facing ingroup pain faces, compared to outgroup faces	Sessa et al. [92]

WIT—"Weapon Identification Task"

ERN—"Error-related negativity potential"

IAT—"Implicit Association Test"

### The impact of racial bias on mental and physical well-being

Racial prejudices observed in the laboratory using the EEG technique have serious effects on our society and can also lead to alterations in psychophysical well-being. In this section we will present some studies in which, using techniques other than EEG, the effects on the health of individuals affected by racial biases were investigated. We believe that this section, although differing from the central corpus of the review in terms of techniques, is complementary to the previous sections as it provides a measure of the actual risks posed by this observable phenomenon in the laboratory on the health of ethnic minority individuals. In fact, a growing body of literature has emphasised that racial discrimination is an important factor potentially causing health problems in ethnic minorities. Berger and Sarnyai, in a recent review [103], provided evidence on the hormonal and neural effects of racial discrimination. It has been observed that chronically elevated cortisol levels and a dysregulated hypothalamic–pituitary–adrenal (HPA) axis mediate the effects of racial discrimination on disease. Furthermore, it was found that racial discrimination can impair prefrontal cortex (PFC) function, showing significant similarities to chronic social stress. In the same year, Paradies et al. [104] conducted a meta-analysis in which they reviewed data from 293 studies published between 1983 and 2013 and examined the relationship between reported racism and mental and physical health outcomes. Overall, the results showed that racism is associated with poorer physical and mental health, including depression, anxiety and psychological distress.

More recently, Korous et al. [105] conducted a meta-analysis on racial discrimination and cortisol levels. The results suggested that the association between racial discrimination and cortisol is complex and that effect sizes varied depending on the method used. Results from another meta-analysis [106] noted that adults’ reported experiences of discrimination are negatively related to mental health and physical health indicators, such as preclinical indicators of disease, treatment utilisation and adherence to medical regimens. Emerging evidence also suggests that discrimination may affect the health of children and adolescents.

Overall, these studies suggest that discriminatory experiences can, like other stressors, trigger physiological responses that negatively affect the maintenance of body homeostasis. In conclusion, exposure to discrimination is an important risk factor for the disease, affecting racial and ethnic minorities significantly.

### Can racial prejudice be reduced?

Recently, a few studies have tried to verify whether racial prejudices can be reduced, which could be of great relevance to society. In Pech and Caspar [107] conducted a study to investigate whether a video game designed to reduce prejudice against minorities in a fictional society has the potential to decrease prejudice against minorities in real life as well. A positive effect of the test game compared to the control game on the attenuation of prejudice against an out-group individual was observed. These results appear very promising as they support the evidence that the use of fictional characters in video games can induce positive changes towards non-fictional individuals.

In another study [108], it was observed that racial prejudice to others' pain is modulated by experience and contact with individuals of a different ethnicity, suggesting that experience reduces racial prejudice. Another work [109] showed that another effective technique to reduce racial prejudice could be group therapy, which, by addressing emotions related to racial prejudice through practical techniques, offers a rich context for dealing with this phenomenon.

Finally, a general but, in our opinion, fundamental suggestion comes from the review of Dotson and Duarte [110]. Here, the authors emphasise the urgency of taking demographic characteristics such as race and ethnicity into account when investigating the neural basis of human cognition. They argue that, although challenging, greater diversity in cognitive neuroscience research is needed to improve reproducibility and to meet the treatment needs of a diverse population.

## Conclusions

The review and study of the ERP components associated with implicit prejudice bias, empathy for pain and recognition of ethnicity via face processing pinpoint the distinct stages involved in the perception and emotional response towards social outgroups. The rationale for the selection of ERP-EEG design as the inclusion criterion for studies in this review is that it offers an objective lens for understanding results frequently drawn from a heterogeneous set of methodologies for studying prejudice, ranging from anthropology and social psychology to cognitive neuroscience. In other words, ERPs allows us to follow the time course of how an observer processes same- or other-race stimuli and forms unfavourable evaluations.

Investigating specific ERP components associated with implicit prejudice allows us to understand the impact of racial bias on attentional orientation, specifically the delay or errors directly attributed to the difficulty in inhibiting implicit or explicit prejudiced responses. This difficulty is due to conflicts on a cognitive and social level, which tend to modulate the participant’s task engagement and his/her future responses.

This review provides an overview of recent studies investigating racial bias using an event-related potential EEG collection and analysis design. The data gathered here focus on the widespread use and application of univariate analysis of evoked potentials for the study of racial bias. According to Ito et al. [111] the use of ERP design in EEG experimentation allows for a greater understanding of how social contexts are perceived and associated with specific stereotypes and behavioural predictions. Thus, ERPs evaluate changes in the neural circuitry and its activity at the level of automatic and controlled processes for the applied tasks. While prejudice is a complex social phenomenon that requires several levels of analysis and a variety of different methods of investigation, EEG and ERP analysis is a relatively modern and efficient tool for testing aspects of social cognition. We hope the combined use of ERP-EEG and both implicit and explicit behavioural measures will yield further insights into our understanding of this phenomenon by facilitating the investigation of more subtle forms of prejudice and of the cognitive processes recruited during social evaluation.

## Data Availability

Not applicable.

## Abbreviations

ACC: Anterior cingulate cortex
EEG: Electroencephalography
ERN: Error-related negativity
Pe: Error-related positivity
ERP: Event-related potential
FPST: First-person shooter task
fMRI: Functional magnetic resonance imaging
IAT: Implicit association test
aI: Anterior insula
IRI: Interpersonal reactivity scale
LPP: Late positive potential
MFG: Medial frontal gyrus
MFN: Medial frontal negativity
OR: Other-race
ORE: Other-race effect
PFC: Prefrontal cortex
SR: Same-race
SPCN: Sustained posterior contralateral negativity
TPJ: Temporo-parietal junction
TOM: Theory of mind
VWM: Visual working memory
WIT: Weapon identification test
